# Maximization of Sulforaphane Content in Broccoli Sprouts by Blanching

**DOI:** 10.3390/foods11131906

**Published:** 2022-06-27

**Authors:** Andrea Mahn, Carmen Elena Pérez, Víctor Zambrano, Herna Barrientos

**Affiliations:** 1 Department of Chemical Engineering, Faculty of Engineering, University of Santiago of Chile, Santiago 9170019, Chile; victor.zambrano@usach.cl; 2 Department of Agro Industrial Engineering, Pontificia Bolivariana University, Cra. 6 No. 97A-99, Montería 230001, Colombia; carmenelena.perez@upb.edu.co; 3 Department of Materials Chemistry, Faculty of Chemistry and Biology, University of Santiago of Chile, Santiago 9170019, Chile; herna.barrientosc@usach.cl

**Keywords:** blanching, sulforaphane, optimization, broccoli sprouts

## Abstract

Broccoli sprouts are a recognized source of health-promoting compounds, such as glucosinolates, glucoraphanin, and sulforaphane (SFN). Maximization of SFN content can be achieved by technological processing. We investigated the effect of blanching conditions to determine the optimal treatment that maximizes sulforaphane content in broccoli sprouts. Broccoli seeds (cv. Traditional) grown under controlled conditions were harvested after 11 days from germination and subjected to different blanching conditions based on a central composite design with temperature and time as experimental factors. Results were analyzed by ANOVA followed by a Tukey test. The optimum conditions were identified through response surface methodology. Blanching increased sulforaphane content compared with untreated sprouts, agreeing with a decrease in total glucosinolates and glucoraphanin content. Temperature significantly affected SFN content. Higher temperatures and shorter immersion times favor glucoraphanin hydrolysis, thus increasing SFN content. The optimum conditions were blanching at 61 °C for 4.8 min, resulting in 54.3 ± 0.20 µmol SFN/g dry weight, representing a 3.3-fold increase with respect to untreated sprouts. This is the highest SFN content reported for sprouts subjected to any treatment so far. The process described in this work may contribute to developing functional foods and nutraceuticals that provide sulforaphane as an active principle.

## 1. Introduction

*Brassicaceae* are a rich source of health-promoting compounds, such as vitamins, minerals, trace elements, polyphenols, and isothiocyanates [1]. Among them, sulforaphane (SFN), the isothiocyanate derived from glucoraphanin (GFN), has generated great attention in recent years because of its outstanding health-promoting properties and its high bioavailability [2]. The heathy effect of SFN relates to its capacity to modulate phase I and phase II detoxifying enzymes preventing some chronic non-transmittable diseases such as cancer and autoimmune diseases [3]. Recent studies report that SFN has cardio protective [4] and obesity preventive effects [5].

SFN is formed from the hydrolysis of GFN, catalyzed by the enzyme myrosinase (E.C. 3.2.1.147) that proceeds after tissue disruption (mastication or grinding) [6]. At neutral pH, the main product is sulforaphane, but at acid pH, with the presence of ferrous ion and epithiospecifier protein (ESP) or nitrile specifier protein (NSP), the hydrolysis products are epithionitriles and nitriles, which have no bioactive properties and can even be toxic.

A possible way to take advantage of the healthy properties of SFN is its incorporation into food matrixes or in dietary supplement formulae. Different strategies have been proposed to incorporate SFN in food matrixes based on processing *Brassicaceae* vegetables as natural sources of SFN. Drying [7], microencapsulation [8], and fermentation [9] are some of the processes studied to increase SFN content and stabilize it in food. Some studies have focused on the effect of hydrothermal processing of broccoli tissues on SFN formation [10,11,12,13]. Pérez et al. [10] found the optimal blanching conditions that inactivate ESP and favor myrosinase activity, resulting in a maximized sulforaphane formation. Mahn et al. [11] reported an increase in SFN concentration in broccoli heads when applying ultrasound-assisted blanching. Ferreira et al. [12] reported a 49% decrease in total glucosinolates content in blanched broccoli by-products in a domestic microwave. Belinska et al. [13] found a significant reduction of isothiocyanates content, by 43%, in freshly harvested broccoli after blanching in water at 85 °C for 3 min.

Thermal processing significantly affects the content of SFN as well as the extractability of bioactive compounds from vegetables. Accordingly, in order to maximize the SFN content in *Brassicaceae*, it is necessary to study the optimal conditions that maximize GFN conversion into SFN and protect SFN from thermal degradation [14]. Processing temperature is a key factor since SFN is a thermolabile molecule that decomposes at temperatures above 40 °C in aqueous media [15].

The main source of GFN, and accordingly SFN, is broccoli. Broccoli sprouts contain up to 100-fold the GFN content found in broccoli florets [16], and broccoli sprouts have higher potential as SFN sources. The effect of processing conditions on SFN content in broccoli sprouts has been poorly studied. Triska et al. [17] investigated different hydrothermal treatments of broccoli sprouts on SFN content, with the main focus on the extraction process. They reported that high temperatures increase SFN formation, but those results were not reproducible at a semi-productive level because of the heterogeneous temperature profile in the batch. Westphal et al. [18] investigated the effect of high-pressure processing on isothiocyanates formation in broccoli sprouts and observed that a 400–600 MPa treatment resulted in the highest SFN formation, probably due to ESP inactivation. Hanschen et al. [19] investigated the effect of temperature and pH on isothiocyanates formation in different *Brassicaceae* tissues, including commercial broccoli sprouts. They found that pH greatly influences isothiocyanate formation but did not report temperature and time conditions that maximize SFN formation.

Currently, there are no studies about the optimization of the blanching process of broccoli sprouts aimed at maximizing SFN formation. The objective of this work was to study the effect of temperature and time in a blanching process on the content of glucosinolates (GSL), GFN, and SFN in broccoli sprouts.

## 2. Materials and Methods

### 2.1. Experimental Design and Statistical Analyses

The experimental design was a central composite with uniform precision, whose factors were blanching temperature and immersion time, with 4 axial points and 5 central points (Table 1). Each experiment was executed in triplicate, in random order. The statistical effect of the experimental factors was determined by ANOVA at a 95% confidence interval followed by the Tukey test. The optimum blanching conditions were determined by the surface response methodology using the polynomial model shown in Equation (1). Here, $\hat{Y}$ is the predicted response; *β*_0_, *β_i_*, *β_ii_*, and *β_ij_* are the regression coefficients for interception, linear, quadratic, and interaction effects, respectively; *k* is the number of independent variables (*k* = 2 in this case); and *X_i_*, *X_j_* are the coded levels of the experimental factors. Data were analyzed with Statgraphics Plus^TM^ (Statistical Graphics Corp., Princeton, NJ, USA).

(1) $$\hat{Y}=\beta_{0}+\overset{k}{\underset{i=1}{\sum }}\beta_{i}\chi_{i}+\overset{k}{\underset{i=1}{\sum }}\beta_{ii}\chi_{i}^{2}+\sum \overset{k}{\underset{i<j=1}{\sum }}\beta_{ij}\chi_{i}\chi_{j}$$

### 2.2. Broccoli Sprouts Culture

Broccoli seeds (*Brassica oleracea* var. *italica* cv. Traditional) were acquired from Monsanto Chile S.A. (Santiago, Chile). After washing with 10% *v*/*v* sodium hypochlorite (Sigma–Aldrich, Schnelldorf, Germany) for 15 min with constant agitation, the seeds were sown in sterile 200 mL Schott flasks containing absorbent paper. Schott Flasks were left in the dark until germination. After germination, the flasks were opened and maintained in a culture chamber at 23 ± 2 °C with a 16/8 light/darkness cycle for 11 days. Finally, sprouts were removed and subjected to the blanching treatments specified by the experimental design.

### 2.3. Blanching

The blanching procedure was similar to that reported by Pérez et al. [10] but using sprouts instead of florets. Briefly, broccoli sprouts (20 g) were put in plastic bags, and the bags were vacuum-sealed and then subjected to the time (3.4–11 min)–temperature (32–88 °C) combinations stated in Table 1, using a thermostatic water bath. After the treatment, the sprouts were set in an ice bath for 5 min and kept at −4 °C until analyses.

### 2.4. Sulforaphane Quantification

Sulforaphane was quantified by reverse-phase HPLC using the method reported by Liang et al. [20]. Samples were ground in a mortar to obtain a homogeneous pulp. One gram of the pulp was extracted two times with 10 mL methylene chloride (Baker, J.T., Center Valley, PA, USA) combined with 0.5 g anhydrous sodium sulfate (Sigma–Aldrich, Schnelldorf, Germany). The organic fraction was evaporated at 30 °C in a rotatory evaporator. The residue was dissolved in acetonitrile (1 mL) (Merck, Darmstadt, Germany) and filtered (0.22 µm membrane filter). Sulforaphane content was determined by HPLC-DAD (Shimadzu, Tokyo, Japan) equipped with a C18 column (5 µm particle size, 250 × 4.6 mm) (Agilent Technologies, Santa Clara, CA, USA). The solvent was 20% acetonitrile in water; the solution was changed linearly for 10 min to reach 60% acetonitrile and maintained at 100% acetonitrile for 5 min to purge the column. The column oven temperature was 30 °C, the flow rate was 1 mL/min, and 20-µL aliquots were used. Sulforaphane was detected at 254 nm and expressed in µmol per g of dry weight (DW) using a sulforaphane standard curve. Analyses were made in triplicate.

### 2.5. Quantification of Total Glucosinolates

Total GSL were quantified as reported by Martínez-Hernández et al. [21]. Broccoli sprouts (50 mg) were pulverized in liquid nitrogen until obtaining a fine powder. The powder was mixed with 1.5 mL 70% methanol (Merck, Darmstadt, Germany) for 30 min at 70 °C. The suspension was centrifuged at 13,000 rpm for 15 min (4 °C). The supernatant was recovered and concentrated in a rotary evaporator (Stuart RE-300, Staffordshire, UK) until dryness. The residue was re-suspended in 1 mL deionized water and then filtered through a 0.45 μm PVDF syringe filter. Detection was performed with an HPLC-DAD equipment (Shimadzu, Tokyo, Japan) provided with a C18 column (5 μm particle size, 250 mm × 4.6 mm) (Agilent Technologies, Santa Clara, CA, USA). The mobile phase consisted of 0.1% trifluoro acetic acid (TFA) (Merck, Darmstadt, Germany) in deionized water and 0.1% TFA in acetonitrile. Column temperature was 30 °C, the flow rate was 1 mL/min, and the injection volume was 20 µL. Detection was made at 227 nm. All determinations were conducted in triplicate. Results were expressed as sinigrin equivalents (µmol/g DW).

### 2.6. Quantification of Glucoraphanin

Glucoraphanin content was determined by reverse-phase HPLC [22]. Broccoli tissue was pulverized; 100 mg of powder were combined with 1.5 mL methanol (70%) in a microcentrifuge tube. The tube was agitated for 30 min in a water bath at 70 °C. Then, the tube was centrifuged (13,000 rpm, 15 min), and the supernatant was recovered and concentrated in a rotatory evaporator at 30 °C. The residue was re-suspended in 1 mL HPLC-grade water. Before injection, the samples were filtered through a 0.22 µm syringe filter. The HPLC equipment was an HPLC-DAD (Shimadzu, Tokyo, Japan) equipped with a reverse-phase C18 column (5µm particle size, 250 × 4.6 mm) (Agilent Technologies, Santa Clara, CA, USA). The mobile phase was TFA 0.1% *v*/*v* in water (buffer A) and TFA 0.1% *v*/*v* in acetonitrile (B). The elution gradient consisted of 5 min of 100% A followed by changing the solution to 94.9% A in a 3 min time lapse. During the next 7 min, the solution was modified to reach 99% B, and finally, 10 min to reach 100% A. The column temperature was 30 °C, and the flow rate was 1 mL/min. Samples were injected in 20 μL aliquots. Glucoraphanin was detected at 226 nm and eluted at 5.0 min. Quantification was performed by comparison with a glucoraphanin standard curve (0.01–7.00 μg). The results were expressed in μmol/g DW. The analyses were made in triplicate.

## 3. Results

Table 1 shows the content of total GSL, glucoraphanin, and sulforaphane achieved after each blanching treatment. The control condition (untreated fresh broccoli sprouts) exhibited the lowest sulforaphane content (16.6 ± 0.4 μmol/g DW), coinciding with the highest total GSL and GFN content. All the blanching treatments produced an increase in sulforaphane content compared with fresh sprouts. In most treatments, this increment was statistically significant, except in treatment T10 (19.4 ± 0.4 µmol/g DW). Conditions T3, T7, and T11 resulted in the highest sulforaphane content, on average, equal to 56.0 ± 1.2 µmol/g DW, with no statistically significant differences between them and differing significantly from all other treatments. In these treatments, the blanching temperature was 60 °C, and blanching time was lower than 8 min.

All conditions produced a significant decrease in glucoraphanin content, agreeing with the increase of sulforaphane observed in all treatments (Table 1). The highest GFN conversion was achieved in the treatments conducted at 60 °C, agreeing with the highest SFN content. The lowest GFN conversion was observed in treatments T5 and T10, conducted at 32 °C and 40 °C, respectively.

Total glucosinolates (GSL) content significantly decreased in all blanching treatments compared with fresh sprouts. The lowest total GSL content occurred in treatments T1–T4, T7, and T11–T13, and the highest GSL content occurred in treatments T5 and T10, as in the case of glucoraphanin.

**Figure 1:**
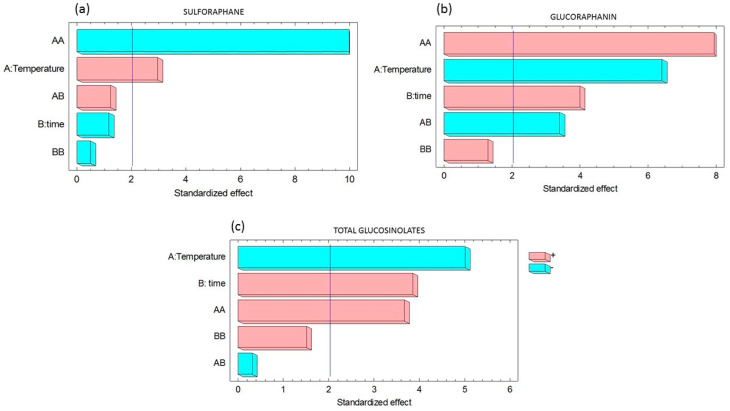
Standardized effects of the experimental factors and their interactions in blanched broccoli sprouts on (**a**) sulforaphane, (**b**) glucoraphanin, and (**c**) total glucosinolates content. Light blue bars indicate positive effects and pink bars indicate negative effects. The bars exceeding the vertical line had a significant effect on the response.

Figure 1 shows the Pareto charts obtained for each response. Figure 1a suggests that only temperature and the interaction of temperature with itself significantly affected the sulforaphane content in blanched broccoli sprouts. Figure 1b indicates that temperature and the interaction of temperature with time had a significant negative effect on glucoraphanin content, whereas time and the interaction of temperature with itself had significant positive effects. Blanching temperature had a significant negative effect on total GSL content, while immersion time and the interaction of temperature with itself had significant positive effects (Figure 1c).

**Figure 2:**
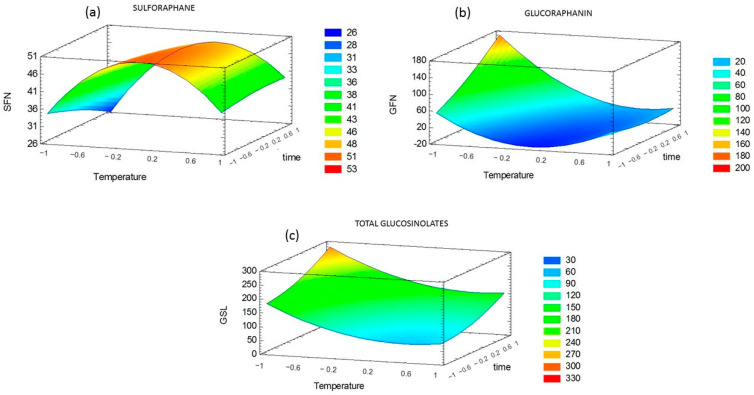
Response surfaces obtained for the content of (**a**) sulforaphane, (**b**) glucoraphanin, and (**c**) total glucosinolates.

The response surfaces (Figure 2) were built based on the regression models presented in Equations (2)–(4). Here, [T] is temperature, [t] is immersion time, [SFN] is sulforaphane, [GFN] is glucoraphanin, and [GSL] is total glucosinolates content.

Equations include only the experimental factors that significantly affected the responses. It must be noted that the model obtained for SFN (Equation (2)) considered only one experimental factor and one interaction, and thus the degrees of freedom were high (d.f. = 34). The model fit gave an R^2^ equal to 76.0% and an R^2^-adjusted equal to 73.9%. Equation (3) gave an adequate adjustment with R^2^ = 77.9% and R^2^ adjusted for degrees of freedom equal to 77.6%. Equation (4) presented the poorest adjustment, with R^2^ and R^2^ adjusted by degrees of freedom equal to 61.3% and 58.0%, respectively, being lower than those obtained for Equations (2) and (3).

[SFN] = 49.419 + 4.042∙[T] − 14.569∙[T^2^] (2)

[GFN] = 9.897 − 43.106∙[T] + 26.868∙[t] + 56.047∙[T]^2^ − 32.383∙[T]·[t] (3)

[GLS] = 120.683 − 59.269∙[T] + 45.680∙[t] + 44104∙[T]^2^ (4)

The blanching conditions that maximized sulforaphane content were determined by the response surface methodology (Figure 2), resulting in 61 °C and 4.8 min. The optimal blanching conditions predicted by the regression model were tested experimentally, resulting in an SFN content of 54.3 ± 0.20 µmol/g DW, representing a 3.3-fold increase with respect to fresh sprouts. Figure 2b shows a minimum glucoraphanin content at 60 °C that agrees with maximum SFN (Figure 2a).

## 4. Discussion

Fresh broccoli sprouts exhibited the lowest sulforaphane content and the highest total GSL and GFN content. This owes to the structural integrity of the vegetal tissue since there was no tissue disruption. This prevents the enzyme myrosinase from getting in contact with its substrate, thus obstructing the hydrolysis.

Blanching at 60 °C for less than 8 min resulted in the highest SFN content. This agrees with [21], who studied the effect of pH and temperature on isothiocyanates formation in broccoli florets subjected to hydrothermal treatments. They found that incubation at 60 °C resulted in the highest sulforaphane content.

Temperature showed a positive effect on SFN content, which means that a temperature increase (i.e., when temperature moves from the low level to the high level of the experimental design) produces an increase in the response. This may be related to an improvement in extractability due to modifications of the vegetable matrix structure induced by temperature. On the contrary, the binary interaction of temperature had a negative effect, suggesting the existence of an optimum temperature that maximizes sulforaphane content within the experimental region. Above this temperature, SFN content decreases, probably due to thermal degradation. This agrees with the response surface built for SFN, as depicted in Figure 2. The factor time had no significant effect on SFN content, probably because the level range was relatively narrow (5–11 min). This result agrees with Pérez et al. [10], who optimized blanching conditions of broccoli florets and detected no significant effect of immersion time when considering a range of 5 to 15 min.

The model fit gave an R^2^ equal to 76.0% and 77.9% for SFN and GFN, respectively, suggesting a reasonable agreement between predicted and experimental values. Thus, both models adequately represent the system behavior.

The optimum conditions that maximize SFN content were blanching at 61 °C for 4.8 min. The optimum temperature coincides with Tríska et al. [17], who concluded that temperature is the key factor in SFN formation in broccoli sprout processing, and that its optimum should range between 60 and 70 °C. Our values match the best temperature reported by [19] for broccoli sprouts (60 °C) and are close to the optimum reported by Pérez et al. [10] for broccoli florets (57 °C for 13 min). Our results also agree with Mahn et al. [11], who reported an increase of SFN content in broccoli florets by 2-fold in comparison with its fresh state when an ultrasound-assisted blanching was applied at 60 °C for 4 min. The optimum conditions predicted by the model were validated experimentally, resulting in a 3.3-fold increase of SFN content with respect to fresh sprouts.

Even though we used around 5 min immersion time to maximize SFN content, we cannot assure that this is the optimum because time had no significant effect on SFN content, and also, this value corresponds to a vertice of the experimental space. It can be speculated that at a shorter immersion time, it would be possible to achieve a similar SFN content, considering the results of Mahn et al. [11].

The SFN content achieved in the present study (~54 µmol/g DW) is the highest reported for broccoli sprouts subjected to any treatment so far. Tríska et al. [17] reported a maximum SFN content of 33 µmol/g of lyophilized sprouts powder subjected to hydrothermal treatment with the addition of exogenous myrosinase. This value represents 61% of the content achieved in the present work. Martínez-Zamora et al. [23] investigated the effect of UV-B treatment on post-harvest SFN content in broccoli sprouts. They reported a maximum SFN content of 6.5 µmol/g DW, representing a 37.5% increase with respect to the SFN content in fresh sprouts. In the present work, we achieved a 330% increment compared to fresh sprouts. The exceptionally high values obtained in this work may be related to the treatment but also to the broccoli cultivar and the culture conditions, which differ from that reported elsewhere [17,23].

Blanching produced a significant decrease in GFN content, accompanied by an increase in SFN. This obeys the enzymatic conversion of glucoraphanin into sulforaphane. However, sulforaphane formation was apparently not equimolar with glucoraphanin consumption, probably due to the conversion of glucoraphanin into other side-products such as sulforaphane nitrile and other thiocyanates. Run T10 GFN had the lowest conversion, equal to 29%, agreeing with Bhat and Vyas [24], who reported only a 20% conversion of glucoraphanin into sulforaphane in broccoli florets (cv. Marathon). However, in the present work, a much higher conversion was achieved in most treatments. This indicates that managing the processing conditions allows for optimizing GFN conversion into SFN in broccoli sprouts. Another explanation for the non-equimolar relation between SFN and GFN is the probable degradation of SFN triggered by temperatures higher than 40 °C [15]. Conversion of glucoraphanin was higher than 99% in runs T1, T3, T4, T7, and T11–T13, greater than the highest GFN conversion reported so far in broccoli florets, equal to 94% [25]. There were no statistically significant differences between these treatments regarding GFN content. The highest GFN conversion was achieved in the treatments conducted at 60 °C, agreeing with the highest SFN content.

The lowest GFN conversion was obtained at 40 °C or lower temperature. This may be related to the optimal temperature of myrosinase. The different broccoli tissues and developmental stages express different myrosinase isoforms, and therefore, the catalytic properties of the enzyme may vary among the different tissues. Myrosinase found in broccoli florets has an optimal temperature of around 40 °C [26], and considering myrosinases from other sources, this temperature may vary between 30 and 70 °C [24]. Broccoli sprout myrosinase has not been isolated yet, and its physicochemical and catalytic properties remain unknown.

Higher blanching temperatures and shorter immersion times favor glucoraphanin hydrolysis, agreeing with the statistical results obtained for sulforaphane. This result agrees with [17]. The significant positive effect of immersion time on GFN content may be related to tissue softening, which facilitates diffusion during the extraction process in analytical determinations.

Total GSL content behaved similarly to glucoraphanin, showing a significant decrease in all blanching treatments compared with fresh broccoli sprouts. However, it comes out from Figure 2c that even though there is a temperature associated with the minimum GSL content, this temperature does not exactly agree with that associated with minimum GFN and maximum SFN content. This may be explained by the different glucosinolates that are present in addition to GFN, which most likely behave differently during blanching.

The regression model obtained for total GSL content resulted in an R^2^ equal to 61.3%, considerably lower than those obtained for SFN and GFN models. This may obey the characteristics of the analytical measurement that detects all GLS, and there is more than one molecule whose dependence on the experimental factors may differ.

In fresh sprouts, the concentration of GFN equals 75% of the concentration of total GSL, reflecting that GFN is the most abundant GSL in broccoli sprouts (75% of total GSL) and confirming that broccoli sprout is the type of biomass with the highest potential as a natural SFN source. The highest SFN content achieved in optimally processed broccoli florets is 4 µmol/g DW [10], while in this work, we achieved around 54 µmol/g DW, 13.5-fold higher than in optimally processed florets.

The results obtained in this work may contribute to the possible industrialization of SFN for its use in the food industry. Even though the SFN content achieved in broccoli sprouts is much higher than that obtained in florets and other broccoli tissues, it is necessary to perform a technical and economic evaluation to decide if using SFN-enriched broccoli sprouts represents a better alternative as a raw material for SFN production than broccoli discards, including florets, leaves, and stalks. Despite the fact that the SFN content achieved in broccoli discards is several folds lower, the cost of the raw material and its production, in the case of sprouts, may have a determinant influence on SFN production cost.

## 5. Conclusions

The conditions that maximize SFN content were blanching at 61 °C for 4.8 min, as predicted by the statistical model. The optimum was experimentally validated, resulting in a 3.3-fold increase in SFN content (~54 µmol/g DW) with respect to fresh sprouts. This is the highest SFN content reported in sprouts subjected to any treatment so far. Higher blanching temperatures and shorter immersion times favor glucoraphanin hydrolysis. GSL content behaved similarly to glucoraphanin, showing a significant decrease in all blanching treatments compared with fresh broccoli sprouts.

## Figures and Tables

**Table 1:** Effect of blanching conditions on total glucosinolates, glucoraphanin, and sulforaphane content in broccoli sprouts. Superscripts indicate significant differences between the samples according to the Tukey test (95% confidence). DW is dry weight.

Run	Temperature (°C)	Time (min)	Sulforaphane (μmol/g DW)	Glucoraphanin (μmol/g DW)	Total GLS (μmol/g DW)
Control	-----	----	16.6 ± 0.4 ^a^	789.0 ± 0.2 ^e^	1042.0 ± 34.6 ^f^
T1	60	7.5	45.8 ± 0.3 ^e^	2.2 ± 0.9 ^a^	103.0 ± 5.5 ^b^
T2	80	5.0	36.5 ± 0.7 ^c^	11.6 ± 3.2 ^a^	12.7± 5.0 ^a^
T3	60	7.5	55.6 ± 0.8 ^f^	3.3 ± 0.2 ^a^	104.0 ± 4.9 ^b^
T4	60	7.5	47.7 ± 3.3 ^e^	2.5 ± 0.5 ^a^	105.0 ± 33.1 ^b^
T5	32	7.5	25.9 ± 0.04 ^b^	138.0 ± 31.0 ^c^	211.0 ± 48.5 ^d^
T6	80	10.0	42.0 ± 0.6 ^d^	51.9 ± 4.0 ^b^	178.0 ± 13.4 ^cd^
T7	60	3.4	57.7 ± 1.6 ^f^	0.6 ± 0.1 ^a^	121.0 ± 3.9 ^bc^
T8	88	7.5	23.6 ± 0.05 ^b^	58.3 ± 4.8 ^b^	151.0 ± 52.8 ^bcd^
T9	40	5.0	23.5 ± 1.0 ^b^	62.9 ± 5.4 ^b^	197.0 ± 18.1 ^cd^
T10	40	10.0	19.4 ± 0.4 ^a^	233.0 ± 23.2 ^d^	383.0 ± 36.4 ^e^
T11	60	7.5	54.8 ± 1.3 ^f^	5.0 ± 0.7 ^a^	121.0 ± 31.9 ^bc^
T12	60	7.5	45.8 ± 1.1 ^e^	4.1 ± 0.1 ^a^	104.0 ± 0.50 ^b^
T13	60	11.0	47.7 ± 0.3 ^e^	4.0 ± 0.7 ^a^	131.0 ± 18.3 ^bcd^
